# Phenol Liquefaction of Waste Sawdust Pretreated by Sodium Hydroxide: Optimization of Parameters Using Response Surface Methodology

**DOI:** 10.3390/molecules27227880

**Published:** 2022-11-15

**Authors:** Shihao Lv, Xiaoli Lin, Zhenzhong Gao, Xianfeng Hou, Haiyang Zhou, Jin Sun

**Affiliations:** College of Materials and Energy, South China Agricultural University, Guangzhou 510642, China

**Keywords:** waste sawdust, alkali–heat pretreatment, liquefaction, response surface methodology, residual content

## Abstract

In this study, a two-step method was used to realize the liquefaction of waste sawdust under atmospheric pressure, and to achieve a high liquefaction rate. Specifically, waste sawdust was pretreated with NaOH, followed by liquefaction using phenol. The relative optimum condition for alkali–heat pretreatment was a 1:1 mass ratio of NaOH to sawdust at 140 °C. The reaction parameters including the mass ratio of phenol to pretreated sawdust, liquefaction temperature, and liquefaction time were optimized by response surface methodology. The optimal conditions for phenol liquefaction of pretreated sawdust were a 4.21 mass ratio of phenol to sawdust, a liquefaction temperature of 173.58 °C, and a liquefaction time of 2.24 h, resulting in corresponding liquefied residues of 6.35%. The liquefaction rate reached 93.65%. Finally, scanning electron microscopy (SEM), Fourier transform infrared spectroscopy (FT-IR), and X-ray diffraction (XRD) were used to analyze untreated waste sawdust, pretreated sawdust, liquefied residues, and liquefied liquid. SEM results showed that the alkali–heat pretreatment and liquefaction reactions destroyed the intact, dense, and homogeneous sample structures. FT-IR results showed that liquefied residues contain aromatic compounds with different substituents, including mainly lignin and its derivatives, while the liquefied liquid contains a large number of aromatic phenolic compounds. XRD showed that alkali–heat pretreatment and phenol liquefaction destroyed most of the crystalline regions, greatly reduced the crystallinity and changed the crystal type of cellulose in the sawdust.

## 1. Introduction

Fossil energy is an important raw material utilized for the production of chemical products [1]. However, the utilization of fossil energy generates a number of greenhouse gases including sulfur oxide, nitrogen oxide, carbon oxide and carbon dioxide, causing air pollution and climate change [2]. Therefore, in order to alleviate energy crises and environmental pollution, researchers need to urgently search for cheap, clean and renewable energy resources [3]. As a natural renewable material with a huge storage capacity, lignocellulosic biomass has attracted increased research attention. It has a rich variety, including crop waste, wood processing residues, and wood product recycling waste, which is considered to be one of the most promising and sustainable alternatives to petroleum for the production of energy, materials and chemicals in the future [4,5].

Several treatment methods such as pyrolysis, liquefaction and gasification have been used to process lignocellulosic biomass to produce biofuels and other valuable chemicals [6,7]. Among them, liquefaction is an effective method for the integrated utilization of lignocellulosic biomass. Liquefaction technologies can be divided into two broad categories, hydrothermal liquefaction and solvent liquefaction [8]. Hydrothermal liquefaction is often carried out under harsh conditions, including high temperatures (200 °C to 400 °C), high pressures (5 MPa to 20 MPa), and a closed environment. Compared with hydrothermal liquefaction, solvent liquefaction can be carried out under mild reaction conditions and is an effective method for the integrated utilization of biomass [9]. Solvent liquefaction usually uses polyols or phenols to liquefy biomass at atmospheric pressure and relatively low temperature. Most often, acid is used as the catalyst and phenol as the solvent in solvent liquefaction, which reflects a high biological conversion rate [10]. The liquefied products are rich in phenolic compounds, which can be used to prepare phenolic resin adhesives after condensation with formaldehyde [11]. However, the liquefied products contain unreacted solvents and acidic catalysts that require recycling or alkali neutralization before preparing adhesives [12].

Further, during the liquefaction, the cellulose and hemicellulose of lignocellulosic biomass are wrapped in highly polymerized polyphenolic structured lignin, which makes it difficult to transform, thus hindering the high-value utilization of lignocellulosic biomass. Targeted pretreatment of lignocellulosic biomass before liquefaction can change the chemical structure and composition of lignocellulose, soften the raw materials, and induce depolymerization and chain breaks [13]. More importantly, the alkaline or acidic conditions of the pretreatment stage can be utilized to meet economic and environmental criteria during the liquefaction, and the pretreated lignocellulosic biomass exhibits better liquefaction performance [14].

Response surface methodology (RSM) is highly effective for the optimal design of a regression model, which is used to resolve problems related to nonlinear data processing [15]. By fitting the regression and plotting the model, the effect of the variables and their interactions on the response variables can be easily evaluated, and the optimal value of the response and the corresponding experimental conditions can be determined. The model obtained by RSM is continuous and can be analyzed continuously for each experimental level during the search for the optimizing experimental conditions, so we can better understand the experimental process [16]. In addition, RSM allows process optimization in a limited number of experimental runs, thus significantly reducing experimental time and costs [17]. 

In this study, a two-step process was used to achieve the effective liquefaction of waste sawdust. The first step entailed the pretreatment of waste sawdust with hot alkali under atmospheric pressure. In the second step, the pretreated sawdust was liquefied using phenol as the liquefying agent. The effects of the mass ratio of sawdust to NaOH on the liquefaction were studied. Based on RSM, the effects of the three experimental variables, namely the mass ratio of phenol to pretreated sawdust, liquefaction temperature, and liquefaction time, and their interactions on the liquefaction effect of waste sawdust were systematically investigated. At the same time, the specific residual content model was established using the design software, which was analyzed in detail to determine the optimal liquefaction conditions. The validity of the model was verified by repeated experiments. Finally, the untreated waste sawdust, pretreated sawdust, liquefied residues and liquefied liquid were characterized and analyzed by scanning electron microscopy (SEM), Fourier transform infrared (FT-IR) spectroscopy, and X-ray diffraction (XRD) to facilitate the high-value utilization of waste sawdust. 

## 2. Results and Discussion

### 2.1. Alkali–Heat Pretreatment—Screening and Optimization Tests

**Figure 1:**
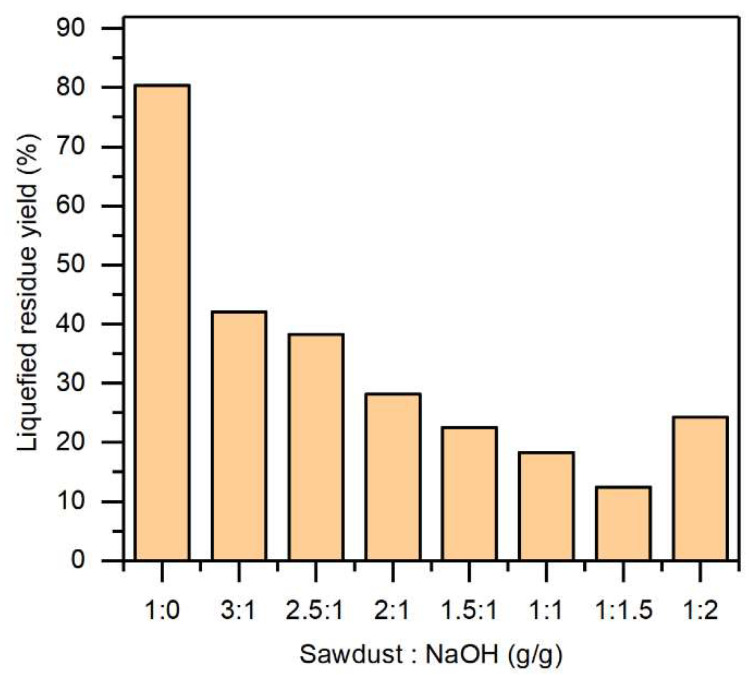
The effect of alkali–heat pretreatment on the liquefied residue yield. The phenol liquefaction temperature was 150 °C, the mass ratio of phenol to pretreated sawdust was 5:1, and the liquefaction time was 2 h.

The effects of the mass ratio of sawdust to NaOH at 140 °C on the liquefied residue yield were studied (Figure 1). It could be seen that the mass ratio of sawdust to NaOH had a significant effect on the liquefied residue yield. The liquefied residue yield decreased initially and then increased as the sawdust to NaOH mass ratio increased. The liquefied residue yield was 12.5% at a mass ratio of 1:1.5. The liquefied residue yield of sawdust without alkali–heat pretreatment under the same liquefaction conditions was 80.4%, which was really high, indicating that the alkali–heat treatment destroyed the internal structure of sawdust, which was conducive to liquefaction. In a certain range, the liquefied residue yield decreased with the reduction in the sawdust to NaOH mass ratio. This was explained by as the amount of NaOH increased, additional wood components were decomposed. Under alkali–heat pretreatment, the carbohydrates are degraded via the peeling reaction of the reducing end groups. Since hemicellulose has a substantially lower molecular weight than cellulose, it is also degraded via hydrolysis in hot alkali, increasing the number of reducing end groups and enhancing the erosion of polymer chains [18,19]. In addition, the presence of NaOH separated the bonds between lignin and carbohydrates, and disrupted the ether bonds between lignin polymers, resulting in the depolymerization and degradation of lignin [20,21]. However, when the mass ratio of sawdust to NaOH was 1:2, the yield of the liquefied residue was slightly increased, indicating that the high proportion of NaOH enhanced the repolymerization of degradation products or inhibited the depolymerization of lignocellulose [22]. 

At a sawdust to NaOH mass ratio of 1:1, the yield of liquefied residue was relatively low. However, when the mass ratio decreased to 1:1.5, the yield of liquefied residue changed slowly. In terms of energy conservation, a satisfactory liquefied residue yield was obtained at a sawdust to NaOH mass ratio of 1:1. Meanwhile, it should be noted that some of the adsorbed NaOH crystal on sawdust during the pretreatment was dissolved during the subsequent liquefaction, which induced cellulose swelling and increased the porosity and specific surface area of the sawdust, thus increasing the accessibility of phenol to sawdust and promoting the degradation of the sawdust [23,24,25]. According to the literature, the liquefied residue of sawdust has a significant impact on the performance of the subsequent fabrication of bio-based phenolic resins [26]. Therefore, it is important to optimize the pretreatment conditions and thus reduce the residual content of liquefaction.

### 2.2. Optimization of Phenol Liquefaction of Waste Sawdust 

#### 2.2.1. Model Fitting

The results of the whole experimental runs are summarized in Table 1. In general, the residual content after phenol liquefaction of waste sawdust was relatively low. Under different liquefaction conditions, the residual content varied from 6.82% to 17.13%, suggesting that phenol liquefaction is an effective and feasible biomass conversion method. The liquefied products can further react with formaldehyde to produce valuable biobased wood adhesive [27]. The lowest residual content was observed in a phenol-to-pretreated sawdust mass ratio of 4.21, a liquefaction temperature of 173.58 °C, and a liquefaction time of 2.24 h. The fitting quadratic multiple regression equation after the exclusion of the insignificant terms for the residual content of waste sawdust is determined based on these data, as shown in Equation (1).

(1) $$\begin{array}{l} R=162.74864\;-\;24.02153A\;-\;1.03750B\;-\;14.186057C\;-\;0.134375\mathrm{BC}\\ +2{.15511A}^{2}+0{.003566B}^{2}+7{.64545C}^{2} \end{array}$$

where R is the residual content (%), A denotes the mass ratio of phenol to pretreated sawdust (P/S), B refers to the liquefaction temperature (°C), and C is the liquefaction time (h). 

In order to further verify the adequacy of the quadratic model, an analysis of variance (ANOVA) was carried out and the results are shown in Table 2. *p*-values less than 0.05 indicate the model variables are statistically significant, and the smaller the *p*-value, the more significant the corresponding coefficient and contribution to the response variable [17]. In this study, the *p*-value of the model was less than 0.0001 and the adjusted R^2^ value was 0.9189. This indicated that the response regression model was highly significant and sufficient for the response variables tested. Further, the “lack of fit f-value” of 2.43 implied that the lack of fit was insignificant relative to the pure error. There was a 17.65% chance of a lack of fit f-value due to noise. The insignificant lack of fit was good, which indicated that the proposed model fitted the data well. Based on the *p*-values, it can be seen that the variables of A, B, C, the interaction term of BC, and the quadratic terms of A^2^, B^2^, C^2^ were significant, indicating that the variables of the reaction were interactive and complex. The significant effect of every single variable on the residual content decreased as follows: A > B > C. The significant effect of interacting variables on residual content decreased in the order of BC > AB > AC. The magnitude of the f-value reflects the importance of each test variable on the index, and the larger f-value indicates the greater importance of the test index [28]. The importance of the influence of the test variables on residual content was as follows: A > B > C, which was identical to the significant effect of every single factor on residual content. In addition, the R^2^ of the selected model was 0.9573, and the fitting degree was more than 95%, indicating that the model effectively reflected the changes in response values. 

**Figure 2:**
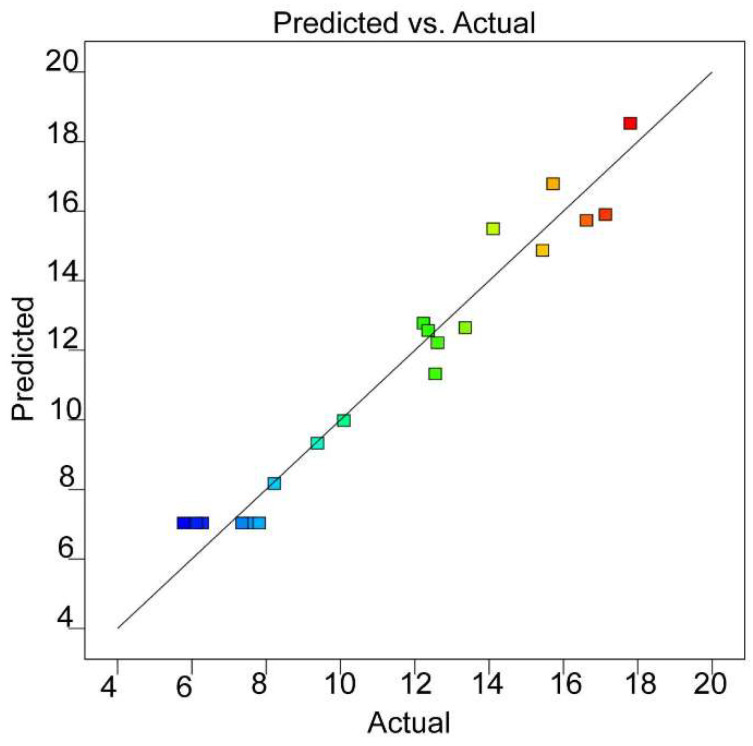
Comparison between the predicted (straight line) and actual response values (points) obtained from the model for the response of residual content.

The comparison between the actual residual content obtained in the experiment and the predicted residual content based on the quadratic model is shown in Figure 2. Generally, each experimental data point should be approximately close to the regression line of the prediction data, which suggests that the estimated effect is true and effective [29]. In this study, the 20 groups of actual experimental values were distributed near the predicted value, which implied a strong correlation between the experimental responses and predicted responses. 

#### 2.2.2. Main Response Surface Plots and Optimization 

The three-dimensional response surface plots and contour plots were used to delineate the effect of independent variables and their interactions on residual content based on the obtained regression equation. The response surface plots of residual content were a function of two specific variables, while the other variable remained at a fixed value. The curve shapes of all response surfaces were upward concave, and all the center and edge points were within the studied range, which indicated that there was an optimal response for the content of liquefied residues. 

**Figure 3:**
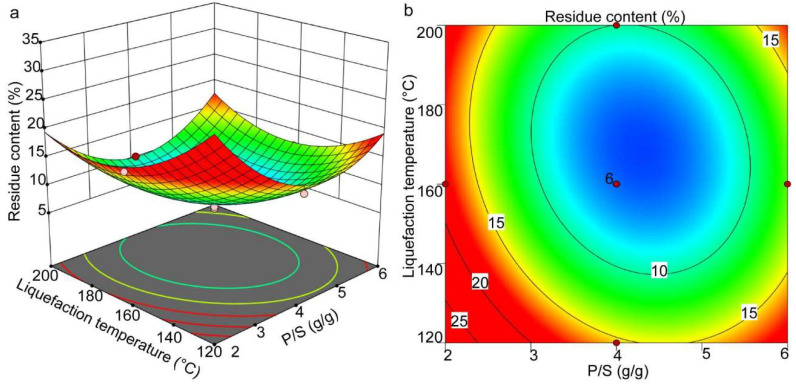
The response surface (**a**) and contour plots (**b**) showing the effects of P/S and liquefaction temperature on the residual content.

The effect of the P/S ratio and the liquefaction temperature on the residual content is shown in Figure 3a,b. It can be seen that P/S and liquefaction temperature significantly affected the residual content. In a specific range, the residual content decreased with the increase in P/S and liquefaction temperature and then increased with the further increased P/S and temperature after reaching the optimal critical point (P/S and temperature of 4.21 and 173.58 °C, respectively). Similar phenomena were observed in other woody materials [30]. The increase in P/S in the range of 2 to 4.21 reduced the residual content, indicating that the P/S values in this range facilitated the liquefaction of sawdust. However, when the P/S increased to a higher value, it negatively affected the sawdust liquefaction. These results suggest that lower P/S values lead to the recondensation of the low-molecular-weight compounds into insoluble residues [31]. An insufficient amount of phenol in the reaction system increases the viscosity of the liquefied products [32]. The P/S value of 4.21 is reasonable to ensure minimum residual content of waste sawdust for adequate liquefaction.

Further, the effect of liquefaction temperature on the residual content showed a similar trend compared with the P/S value. With the increase in reaction temperature, the residual content decreased first and then increased above 173.58 °C which is explained by the incomplete bond cleavage of different components of waste sawdust at lower temperatures due to less violent reaction conditions. In addition, reactions, such as hydrolyzation and depolymerization, resulting in smaller molecules could not be completed [33]. As the temperature increases, the glycosidic bonds break, leading to dehydration and decarboxylation, resulting in the cleavage of large molecules into smaller fragments [34], and a decrease in the residual content. However, with the further increase in temperature (>173.58 °C), the residual content increased gradually. This was mainly because of the unstable components in the liquefied product at high temperatures [35], and a series of complex reactions resulting in the formation of a large number of liquefied residues. 

**Figure 4:**
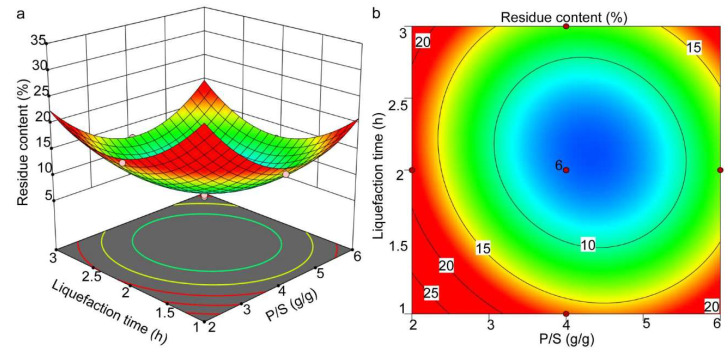
The response surface (**a**) and contour plots (**b**) showing the effects of P/S and liquefaction time on the residual content.

The comprehensive effects of the P/S ratio and liquefaction time on the residual content at a constant temperature are depicted in Figure 4a,b. It can be observed that the critical transition point was established when the P/S value was 4.21 and the liquefaction time was 2.24 h. With the increase in liquefaction time from 1 h to 2.24 h, the residual content gradually decreased to less than 5.6%. However, the residual content increased if the liquefaction time continued to extend. In the initial stage of the reaction, the degradation reaction played a dominant role, which decreased the residual content [32]; the residual content decreased. With the further extension of the reaction time, the polycondensation reaction and the degradation reaction among the liquefaction products gradually reach the balance until they took the dominant role, which led to the increase in the residue content [36].

**Figure 5:**
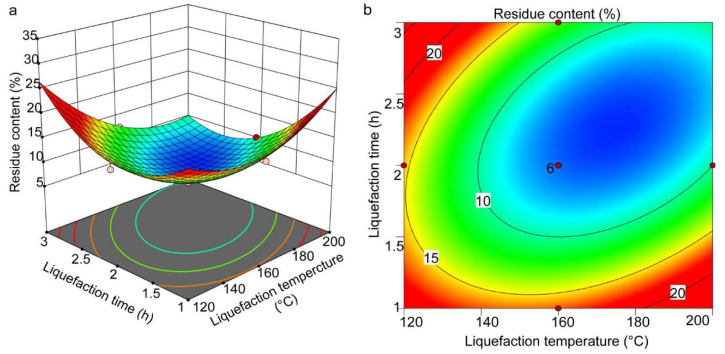
The response surface (**a**) and contour plots (**b**) showing the effects of liquefaction temperature and liquefaction time on the residual content.

Figure 5a,b show the effects of liquefaction time and liquefaction temperature on the residual content at a constant P/S value. It can be seen that the effect of liquefaction time was closely related to the liquefaction temperature. Specifically, the variation in residual content over time was more pronounced at higher temperatures than at lower temperatures. At a constant liquefaction temperature (greater than 173.58 °C), when the liquefaction time increased from 1 h to 2.24 h, the residual content decreased significantly. Further extension of time to 2.6 h or longer resulted in an almost constant residual content or a slight increase. Similarly, compared with the shorter reaction times, the change in residual content with temperature was more significant under the longer reaction times. At a constant reaction time (greater than 2.24 h), the residual content decreased significantly with the temperature increase from 100 °C to 180 °C, followed by equilibrium and then a slight increase. This also confirmed that heating at 173.58 °C for 2.24 h facilitated waste sawdust liquefaction. 

To demonstrate the accuracy and reliability of the optimal liquefaction conditions determined from the fitted model, the experiment was repeated three times using the same method based on the predicted optimal point and the average value was calculated. The actual residual content obtained experimentally was 6.35%, while the residual content predicted based on Equation (1) was 6.15%. The results show that the experimental value agrees well with the model prediction, with an error of only 3.25%. It is thus clear that RSM can be used to optimize the process of waste sawdust phenol liquefaction. The optimal liquefaction occurred at a P/S ratio of 4.21, liquefaction temperature of 173.58 °C, and liquefaction time of 2.24 h.

### 2.3. SEM Analysis

**Figure 6:**
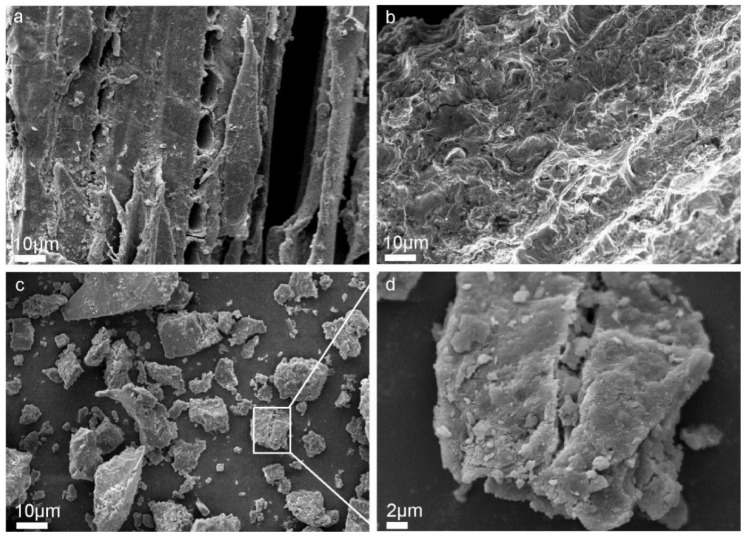
SEM micrographs of untreated sawdust (**a**), alkali–heat pretreated sawdust (**b**) liquefied residues (**c**,**d**).

SEM was used to assess the structural and morphological changes of sawdust after treatment (Figure 6). The surface of untreated sawdust was continuous and smooth, and the fibrous structure was relatively complete (Figure 6a), in which lignin was the encrusting material connecting the fibers and hemicellulose was the filling material distributed in the microfibers of the cell wall [37]. The pretreated sawdust (Figure 6b) revealed many microfibril aggregates with a rougher and more wrinkled surface due to the activation of lignocellulose with alkali, resulting in partial structural degradation [38]. Further, the rough surface of pretreated sawdust increased the specific surface area, which increased the accessibility of phenol during the subsequent liquefaction. Following phenol liquefaction, the structure of the pretreated sawdust was severely damaged, with a lower degree of polymerization and loose and irregular texture (Figure 6c,d), due to the surface coke generated by the condensed lignin and cellulose [39]. 

### 2.4. FT-IR Analysis

**Figure 7:**
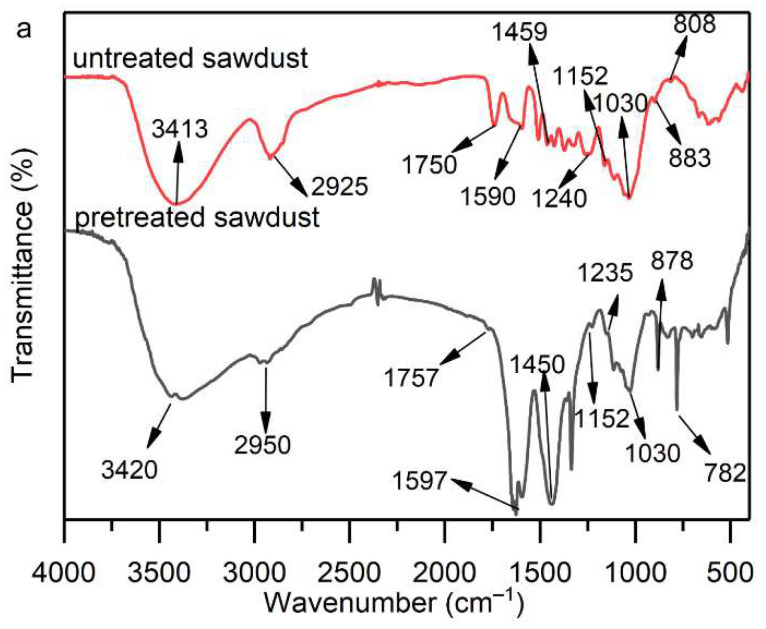

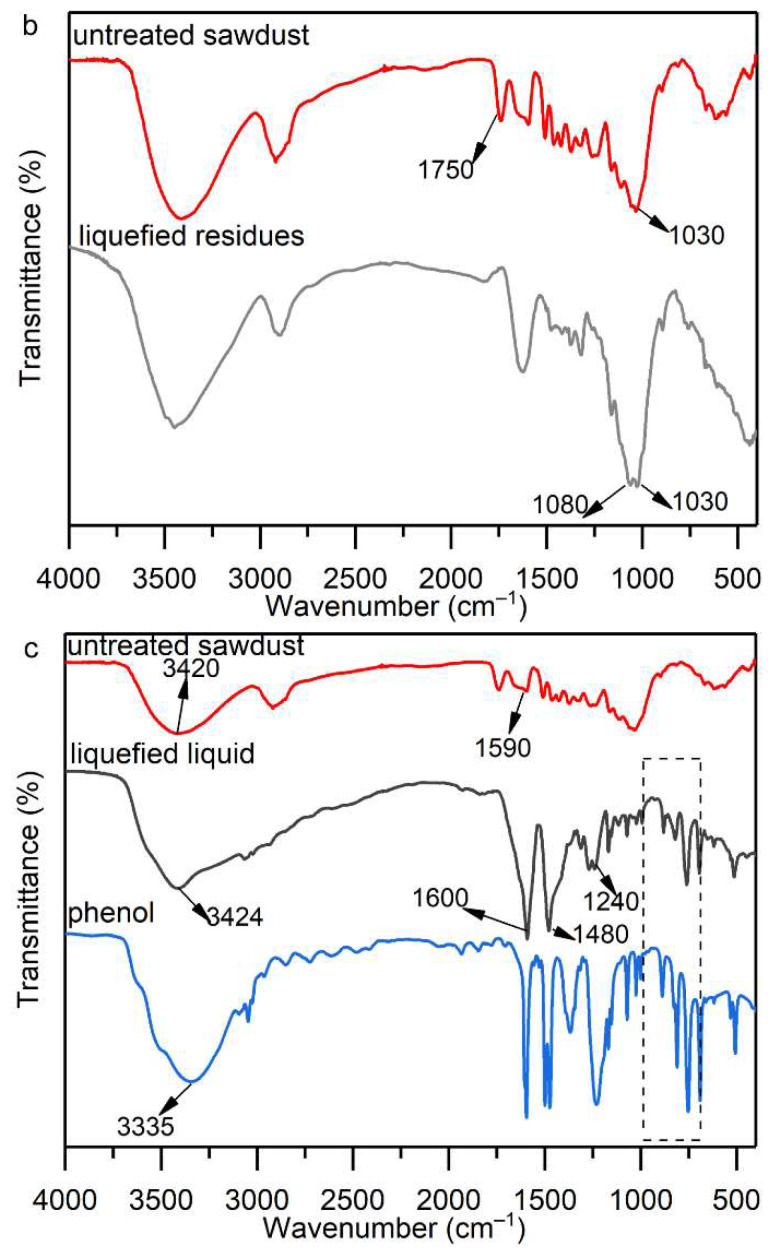
FT-IR spectra of untreated sawdust and pretreated sawdust (**a**), untreated sawdust and liquefied residues (**b**), untreated sawdust, liquefied liquid and phenol (**c**).

The functional groups present in the untreated sawdust, pretreated sawdust, liquefied residues, and liquefied liquid were investigated by FT-IR. For untreated sawdust (Figure 7a), the broad peak at around 3400 cm^−1^ indicated O−H stretching vibrations [40]. The peak at 2925 cm^−1^ corresponded to the stretching vibration of C−H. The peaks between 1400 cm^−1^ and 1600 cm^−1^ are attributed to the aromatic skeleton in lignin [41]. Several peaks from 600 cm^−1^ to 900 cm^−1^ represent the characteristic peaks of aromatic monomers. Compared with untreated sawdust, the peak intensity of pretreated sawdust at 2925 cm^−1^ was significantly weakened, which suggested that pretreatment peels off most of the methylene groups in the aliphatic acid methylene group [42]. In addition, the weakening of the peaks at 1750 cm^−1^, 1240 cm^−1^, 1152 cm^−1^ and 1030 cm^−1^ indicates the breakage of cellulose glycosidic bonds and hemicellulose chains, suggesting that large amounts of hemicellulose and part of cellulose were degraded by hydrolysis and peeling reactions during the pretreatment [43]. Compared with untreated sawdust, the peaks between 1400 cm^−1^ and 1600 cm^−1^ and from 600 cm^−1^ to 900 cm^−1^ were enhanced, indicating that pretreatment promoted the decomposition of lignin into aromatic monomers and increased the formation of low-molecular-weight oligomers. It was noteworthy that the new peak of pretreated sawdust around 1332 cm^−1^ might correspond to the characteristic peak of NaOH.

After the liquefaction reaction, the disappearance of the peak at 1750 cm^−1^ for the liquefied residues (Figure 7b) indicated that the hemicellulose was further decomposed during the liquefaction. The strong peak of C−O at 1030 cm^−1^ associated with liquefied residues demonstrated the presence of a large amount of lignin and its derivatives remaining in the liquefied residues [39]. A strong peak at 1080 cm^−1^ attributed to the C–O peak in cellulose, indicated the presence of unliquefied cellulose in the liquefied residues. The peak enhancement at 1600 cm^−1^ and 1480 cm^−1^ indicated the presence of large amounts of aromatic compounds and their derivatives from lignin in liquefied liquid (Figure 7c) Similar phenomena were reported in previous studies [44,45]. The peak of the liquefied liquid around 1240 cm^−1^ may be due to the C–O stretching of the phenol or ester [46]. In addition, other peaks in the range of 600 cm^−1^ to 1000 cm^−1^ in the spectra of the liquefied liquid were attributed to C–H bending vibrations of aromatic hydrocarbons. The enhancement of these peaks can also be explained by the presence of a large number of phenolic compounds in the liquefied liquid.

### 2.5. XRD Analysis

Waste sawdust showed two characteristic peaks of type I lignocellulose at 16.1° and 22.5°, which correspond to the lattice planes of (101) and (002), respectively [47]. The two peaks were both shifted after alkali–heat pretreatment due to the transformation of some cellulose crystals [48]. The Crystallinity Index (*I*_Cr_) of the pretreated samples (18.21%) was reduced by 59.11% compared with untreated natural waste sawdust (44.53%) because a large number of hydrogen bonds between and within the cellulose were destroyed, resulting in the disruption of the crystal structure [49]. Besides, several small peaks between 28° and 50°resulted from the NaOH crystals rearranged in the hierarchical sawdust structures [50].

**Figure 8:**
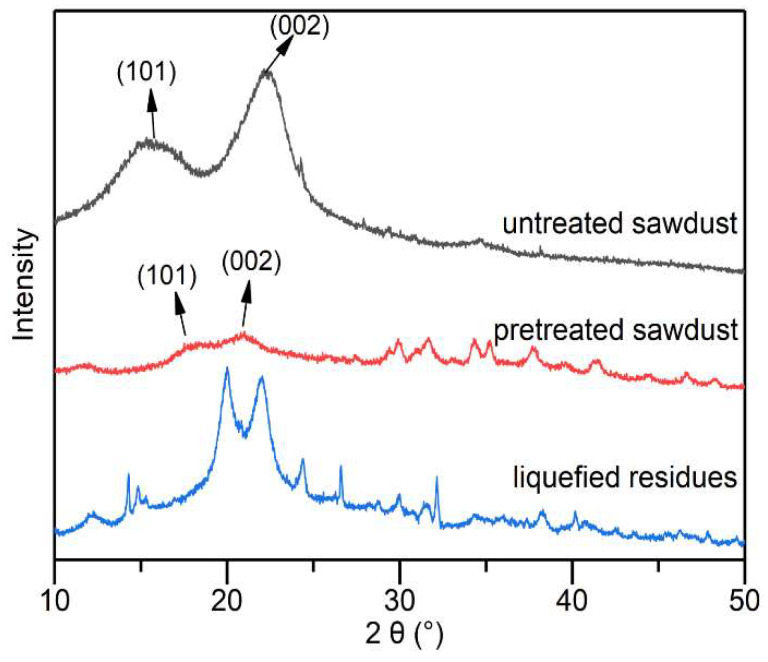
X-ray diffraction of untreated sawdust, alkali–heat pretreated sawdust and liquefied residues.

The XRD spectra of the liquefied residues exhibit many diffraction peaks (Figure 8), indicating that the liquefied residues contained several crystalline substances, which were difficult to liquefy. This was consistent with the results of previous studies [51]. The liquefied residues showed two peaks of type II cellulose at positions 20° and 22°, suggesting the synergistic effect of alkali–heat pretreatment and phenol liquefaction, resulting in altered cellulose crystals. Type II cellulose is stabler and less susceptible to liquefaction, which also explains its presence in the liquefied residues. A sharp peak at 26.5° was due to the formation of carbonaceous structures, such as graphite, during liquefaction. The peak at about 32.2° was attributed to oxidized lignin [52], which corresponded to the results of the FT-IR analysis. 

## 3. Materials and Methods

### 3.1. Materials

The waste sawdust with a size of 20 mesh was generously provided by the carpentry factory located at the South China Agricultural University. The chemical and elemental composition of the sawdust is shown in Table 3. Phenol (99%) and NaOH were purchased from Aladdin Reagent Company, Shanghai, China.

### 3.2. Pretreatment

**Figure 9:**
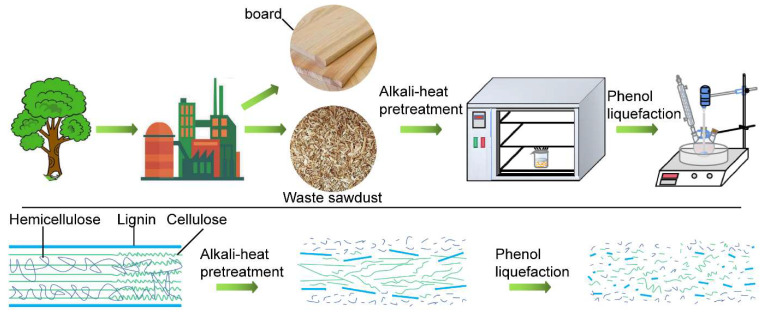
Overall flow chart of two step liquefaction of waste sawdust.

The sawdust was dried to absolute dryness in a drying oven at 103 °C ± 2 °C. The pretreatment was performed in a glass beaker (150 mL) with tinfoil, and heated in an oven. Briefly, 5 g dry sawdust, 25 g distilled water and some NaOH were loaded into the reaction vessel and mixed thoroughly. The mass ratios of dry sawdust to NaOH were 3:1, 2.5:1, 2:1, 1.5:1, 1:1, 1:1.5, and 1:2. The beaker was then transferred to a drying oven and heated to a fixed temperature (140 °C) until the distilled water was dried (about 280 min). At the end of the pretreatment, the beaker was stored in a glass desiccator for subsequent liquefaction experiments. The overall flow chart of the two step-liquefaction is presented in Figure 9.

### 3.3. Phenol Liquefaction

The pretreated sawdust and phenol were loaded into a reactor equipped with a stirrer and a condenser. The whole assembly was immersed into an oil bath preheated to a given liquefaction temperature for a certain reaction time under continuous stirring. Then, the resulting reaction mixture was diluted and dissolved in hot distilled water (50 mL). The insoluble residues were separated by filtration with a G2 glass filter (30–50 μm) under vacuum (0.095 MPa). The filtrate was subjected to rotary evaporation under a vacuum at 70 °C to remove water. The residues were dried in an oven at 103 °C ± 2 °C to constant weight. All experiments were performed in triplicates and the average value was taken. All product yields were calculated using the following equations.

(2) $$\mathrm{Liquefied}\;\mathrm{residue}\;\mathrm{yield}\;(\mathrm{wt}\;\%)=\frac{\mathrm{Weight}\;\mathrm{of}\;\mathrm{Liquefaction}\;\mathrm{Residue}}{\mathrm{Weight}\;\mathrm{of}\;\mathrm{Sawdust}}\times 100$$

(3) Liquefaction yield (wt %)=(1−Liquefied residue yield)× 100

### 3.4. Experimental Design and Process Optimization Using Response Surface Methodology (RSM)

To investigate the effect of independent variables on the response value (residual content) and optimize the liquefaction conditions, experiments with three key variables and five levels were designed using the Box–Behnken design. The Design-Expert 12.0.3.0 software was used to design experiments, perform the statistical analysis, and create the regression model. Based on extensive pre-experiments, the independent variables that significantly influenced the residual content and the right levels were selected. The three independent variables were the mass ratio of phenol to pretreated sawdust (A), liquefaction temperature (B), and liquefaction time (C). The range of each value was chosen in the range of 2 to 6, 120 to 200 °C, and 1 to 3 h, as shown in Table 4. The experimental design included a total of 20 experiments, corresponding to eight factor points, six axial points, and six center point replications to ensure the accuracy of the experiment. 

What is more, the complete design matrix and actual residue are shown in Table 1, and the experimental data were analyzed using Design Expert 12.0.3.0. The test data were fitted to the following second order polynomial equation, as shown in Equation (4). The analysis of variance (ANOVA) and significance test was carried out for the residual content under different conditions to evaluate the quality of the model fitting. All these experiments were carried out in random order.

(4) $$Y=\beta_{0}+\sum_{i=1}^{n}\beta_{i}X_{i}+\sum_{i=1}^{n}\beta_{ii}X_{i}^{2}+\sum_{i=1}^{n}\sum_{j=i+1}^{n}\beta_{ij}X_{i}X_{j}$$

where *Y* is the response function (residue content), *β*_0_ is the model intercept, *β_i_*, *β_ii_* and *β_ij_* represent coefficients of linear, quadratic, and interaction terms, respectively.

### 3.5. Characterization 

#### 3.5.1. Chemical and Elemental Composition Analysis

The chemical composition of sawdust was performed according to the Van Soest method [53]. Briefly, the neutral detergent fiber (NDF), acid detergent fiber (ADF), and acid detergent lignin (ADL) were prepared in turn by deterging waste sawdust sequentially with neutral detergent reagent, acid detergent reagent and 72% H_2_SO_4_. Another amount of dry sawdust was put in a muffle furnace at 600 °C for 6 h, and the ash content was calculated by the weight difference. The difference values between ADF and NDF, ADF and ADL, ADL and ash were considered as contents of hemicellulose, cellulose and Klason lignin, respectively [49]. The elemental analysis of sawdust was performed with an Elemental Analyzer (Vario EL cube, Elementar, Hanau, Germany). The chemical and elemental composition of the sawdust was analyzed three times and the average values were taken separately.

#### 3.5.2. Scanning Electron Microscope (SEM) Analysis 

The morphology of untreated sawdust, alkali–heat pretreated sawdust and liquefied residues were analyzed via SEM (EVO MA 15, ZEISS, Oberkochen, Germany). The working voltage was 10 kV.

#### 3.5.3. Fourier Transform Infrared Spectroscopy (FT-IR) Analysis 

FT-IR instrument (Vertex 70, Bruker, MA, USA) was used to analyze the functional groups in the samples (untreated sawdust, alkali–heat pretreated sawdust, liquefied residues and liquefied liquid). The sample was diluted nicely in KBr. The sample was scanned 32 times in the range of 400 cm^−1^ to 4000 cm^−1^ at a resolution of 4 cm^−1^. Background spectra were recorded before every sampling. 

#### 3.5.4. X-ray Diffraction (XRD) Analysis

Samples ground to powder (100 mesh) were analyzed by X-ray diffraction (xrd-6000, Shimadzu, Kyoto, Japan) with an AlKα radiation source at 40 kV. The scanning range was 5 to 50° with a step of 0.02° at a scanning rate of 10°/min. 

## 4. Conclusions

In this study, a facile method was used to liquefy waste sawdust via a two-step method at a significant liquefaction rate. The alkali–heat pretreatment was optimized by a temperature of 140 °C and a 1:1 mass ratio of sawdust to NaOH, resulting in a 4.2-fold higher liquefaction rate than that of untreated sawdust. Based on the response model established by RSM, it was found that the P/S ratio was the most important variable affecting the liquefaction yield. A P/S ratio of 4.21, a liquefaction temperature of 173.58 °C, and a liquefaction time of 2.24 h were the optimal conditions for phenol liquefaction of pretreated sawdust, resulting in corresponding liquefied residue yield of 6.35%. Thus, the liquefaction rate reached 93.65%. Based on SEM, FTIR, and XRD analyses, alkali–heat pretreatment is essential for subsequent phenol liquefaction. 

## Figures and Tables

**Table 1:** Liquefaction variables and levels.

	Factors			
Order	Mass Ratio of Phenol to Pretreated Sawdust, P/S	Liquefaction Temperature, T (°C)	Liquefaction Time, t (h)	Residual Content, R (%)
1	3	140	1.5	17.13
2	5	140	1.5	12.55
3	3	180	1.5	15.44
4	5	180	1.5	12.61
5	3	140	2.5	16.62
6	5	140	2.5	13.36
7	3	180	2.5	9.38
8	5	180	2.5	8.22
9	2	160	2	17.8
10	6	160	2	12.23
11	4	120	2	14.11
12	4	200	2	10.09
13	4	160	1	15.72
14	4	160	3	12.36
15	4	160	2	7.58
16	4	160	2	7.35
17	4	160	2	7.81
18	4	160	2	6.28
19	4	160	2	5.78
20	4	160	2	6.12

**Table 2:** Results of the analysis of variance for residual content.

Source	Sum of Squares	df	Mean Square	*F* Value	*p* Value	
Model	284.38	9	31.60	24.91	<0.0001	significant
A	32.98	1	32.98	26.00	0.0005	
B	30.39	1	30.39	23.96	0.0006	
C	17.79	1	17.79	14.02	0.0038	
AB	1.85	1	1.85	1.46	0.2546	
AC	1.12	1	1.12	0.8811	0.3700	
BC	14.45	1	14.45	11.39	0.0071	
A^2^	116.78	1	116.78	92.07	<0.0001	
B^2^	51.15	1	51.15	40.33	<0.0001	
C^2^	91.85	1	91.85	72.42	<0.0001	
Residual	12.68	10	1.27			
Lack of fit	8.98	5	1.80	2.43	0.1765	not significant
Pure error	3.70	5	0.7404			
Cor total	297.06	19				

**Table 3:** Element and chemical composition of sawdust and the values were given on a dry basis.

Polymer Mass Fraction (%)			Element Mass Fraction (%)					Ash (%)
Cellulose	Hemicellulose	Klason Lignin	C	H	O	S	N	
42.11	24.32	28.06	46.77	5.97	46.52	0.00	1.01	1.6

**Table 4:** Liquefaction variables and levels.

Variables	Level					
	Code	−2	−1	0	1	2
Mass ratio of phenol to pretreated sawdust, P/S	A	2	3	4	5	6
Liquefaction temperature, T (°C)	B	120	140	160	180	200
Liquefaction time, t (h)	C	1	1.5	2	2.5	3

## Data Availability

Not applicable.
